# Relative percentage and zonal distribution of mesenchymal progenitor cells in human osteoarthritic and normal cartilage

**DOI:** 10.1186/ar3320

**Published:** 2011-04-15

**Authors:** David Pretzel, Stefanie Linss, Steffen Rochler, Michaela Endres, Christian Kaps, Saifeddin Alsalameh, Raimund W Kinne

**Affiliations:** 1 Experimental Rheumatology Unit, Department of Orthopedics, University Hospital Jena, Klosterlausnitzer Str. 81, Eisenberg, D-07607, Germany; 2 Laboratory of Organic and Macromolecular Chemistry, Friedrich-Schiller-University Jena, Humboldtstr. 10, Jena, D-07743, Germany; 3 TransTissue Technologies, Charitéplatz 1/Virchowweg 11, Berlin, D-10117, Germany; 4 Department of Medicine, Division of Rheumatology & Clinical Immunology, P.O. Box 2951, Mafraq Hospital, Abu Dhabi, United Arab Emirates

## Abstract

**Introduction:**

Mesenchymal stem cells (MSC) are highly attractive for use in cartilage regeneration. To date, MSC are usually recruited from subchondral bone marrow using microfracture. Recent data suggest that isolated cells from adult human articular cartilage, which express the combination of the cell-surface markers CD105 and CD166, are multi-potent mesenchymal progenitor cells (MPC) with characteristics similar to MSC. MPC within the cartilage matrix, the target of tissue regeneration, may provide the basis for in situ regeneration of focal cartilage defects. However, there is only limited information concerning the presence/abundance of CD105^+^/CD166^+ ^MPC in human articular cartilage. The present study therefore assessed the relative percentage and particularly the zonal distribution of cartilage MPC using the markers CD105/CD166.

**Methods:**

Specimens of human osteoarthritic (OA; *n *= 11) and normal (*n *= 3) cartilage were used for either cell isolation or immunohistochemistry. Due to low numbers, isolated cells were expanded for 2 weeks and then analyzed by flow cytometry (FACS) or immunofluorescence in chamber slides for the expression of CD105 and CD166. Following immunomagnetic separation of CD166^+^/^- ^OA cells, multi-lineage differentiation assays were performed. Also, the zonal distribution of CD166^+ ^cells within the matrix of OA and normal cartilage was analyzed by immunohistochemistry.

**Results:**

FACS analysis showed that 16.7 ± 2.1% (mean ± SEM) of OA and 15.3 ± 2.3 of normal chondrocytes (n.s.) were CD105^+^/CD166^+ ^and thus carried the established MPC marker combination. Similarly, 13.2% ± 0.9% and 11.7 ± 2.1 of CD105^+^/CD166^+^cells, respectively, were identified by immunofluorescence in adherent OA and normal chondrocytes. The CD166^+ ^enriched OA cells showed a stronger induction of the chondrogenic phenotype in differentiation assays than the CD166^+ ^depleted cell population, underlining the chondrogenic potential of the MPC. Strikingly, CD166^+ ^cells in OA and normal articular cartilage sections (22.1 ± 1.7% and 23.6% ± 1.4%, respectively; n.s.) were almost exclusively located in the superficial and middle zone.

**Conclusions:**

The present results underline the suitability of CD166 as a biomarker to identify and, in particular, localize and/or enrich resident MPC with a high chondrogenic potential in human articular cartilage. The percentage of MPC in both OA and normal cartilage is substantially higher than previously reported, suggesting a yet unexplored reserve capacity for regeneration.

## Introduction

Over the past decades, mesenchymal stem cells/mesenchymal progenitor cells (MSCs/MPCs) have been discovered in almost all tissues, including peripheral blood, bone marrow, muscle, fat, pancreas, skin, and nervous system, and, interestingly, in cartilage [1-5]. Although some of the above non-cartilage MPCs are accessible more easily and in higher numbers, MPCs resident in cartilage may be particularly suitable for novel *in situ *regeneration strategies, including cell-free implant materials with or without bioactive components [6-8].

Compared with numerous reports on classic sources such as bone marrow, there is only limited information about the presence of MPCs with defined biomarkers in human articular cartilage [2-5,9]. Despite extensive efforts, the emerging field of stem cell research still strives to establish well-defined marker constellations, which unambiguously describe the typical stem/progenitor cell phenotype. In the case of cartilage MPCs, most approaches use markers already successfully described for other tissues (for example, bone marrow). However, MPCs isolated from different tissues may not show the same immunophenotype. Possible strategies to identify MPCs by their functional characteristics range from their colony-forming efficacy/clonal growth [10,11] or differential adhesion to fibronectin [12] to the differential uptake of cell-penetrating dyes [13] or their ability to grow out of cartilage tissue [9]. Alternatively, the expression of typical membrane-associated proteins can be employed for the selection of MPCs. These include the expression of Notch-1 [10,14] or triple positivity for CD44/CD151/CD49c [3] or CD9/CD90/CD166 [4]. In addition, co-expression of CD105 and CD166 has been suggested to identify not only bone marrow-derived but also cartilage MPCs [5,15]. CD105, also known as endoglin, is a membrane glycoprotein located on the cell surface. Besides functioning as part of the transforming growth factor (TGF)-beta receptor complex, it affects cell morphology and migration and participates in developmental processes. It has been found on a variety of cells such as endothelial cells, activated macrophages, fibroblasts, smooth muscle cells, and the vast majority of human cartilage chondrocytes [5,16]. The activated leukocyte cell adhesion molecule (ALCAM), also called CD166, is a member of the immunoglobulin (Ig) superfamily and a ligand for CD6, which is involved in T-cell adhesion and co-stimulation [17]. Besides being expressed on thymic epithelial cells, activated T cells, B-lymphocytes, and monocytes, CD166 is expressed on a subpopulation of human cartilage cells [5,18].

Even though the presence of CD105^+^/CD166^+ ^MPCs in adult human cartilage has been reported before, there is no information about their localization within the cartilage matrix. This report is the first to describe their distribution within adult human articular cartilage. This knowledge may have implications for currently evolving concepts in cartilage repair.

## Materials and methods

### Cartilage preparation

Human osteoarthritis (OA) cartilage was obtained from the knee joints of 11 patients who had high-grade OA and who underwent total joint replacement surgery in the Orthopedic Clinic, Waldkrankenhaus 'Rudolf Elle' GmbH, Eisenberg, Germany (Table 1). Clinical and radiological criteria were used for the classification of OA; patients with systemic inflammatory diseases such as rheumatoid arthritis were excluded. Normal cartilage was obtained from the femoral condyles and tibial plateaus of healthy organ donors or at autopsy from donors with no known history of joint disease. The study was approved by the ethics committees of the University Hospital Jena/Charité-University Medicine Berlin, and all patients gave their informed consent.

**Table 1:** Clinical and radiological characteristics of the patients at the time of total joint replacement surgery

Patient	CRP, mg/L	ESR, mm/hour	ICRS grading score	Kellgren-Lawrence grading scale
1	0.3	9	3A	3
2	2.0	7	3A	3
3	1.2	5	3A	3
4	6.3	9	2B	2
5	5.4	19	2A	1
6	0.3	6	3A	3
7	8.1	23	3A	3
8	3.0	12	2A	2
9	10.0	9	3C	4
10	1.7	12	3B	4
11	4.3	12	3B	4

For the parameters of age, C-reactive protein (CRP) (normal range <5 mg/L), and erythrocyte sedimentation rate (ESR) (normal range <30 mm/hour), individual values are presented; x-ray grading of osteoarthritis was performed on the basis of the Kellgren-Lawrence scale [38], and macroscopic cartilage injury was graded according to the Hyaline Cartilage Lesion Classification System of the International Cartilage Repair Society (ICRS) [39]. The patient cohort consisted of 3 male and 8 female cartilage donors with a mean age of 69.6 ± 1.6 years and 3 left/8 right affected knee joints.

**Figure 1:**
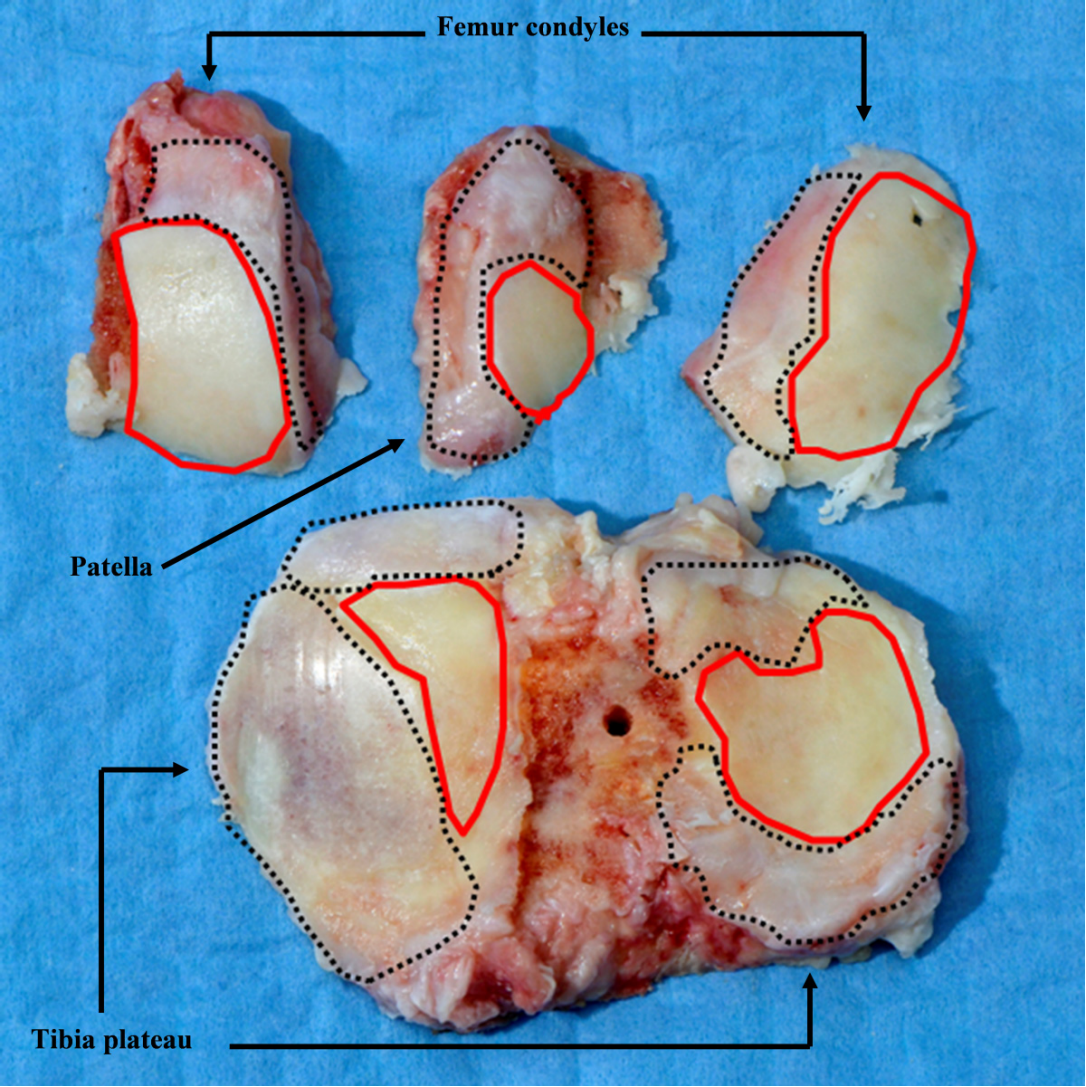
**Image of a representative osteoarthritis cartilage/bone specimen obtained during a total joint replacement surgery**. Locations used for the harvesting of cartilage are indicated by red lines, and excluded regions are encircled with black dotted lines.

For immunohistological analysis, osteochondral samples were prepared using a handsaw, directly fixed in 4% paraformaldehyde in phosphate-buffered saline (PBS), and then subjected to paraffin embedding. For isolation of OA cells, cartilage with mild to moderate macroscopic alterations was carefully harvested with a scalpel from the femoral condyles, the tibial plateaus, and the patella of the knee joints in order to maximize the cell yield (Figure 1). In the case of cartilage from healthy donors, all normal-appearing tissue was harvested. To standardize the procedure and to avoid contamination of the chondrocytes with bone marrow cells, the subchondral lamella was left intact in all cases. Cartilage slices were directly transferred into a dish containing PBS supplemented with antibiotics (100 units/mL penicillin and 100 μg/mL streptomycin).

### Isolation and culture of cells from cartilage

Cartilage slices were washed twice in PBS supplemented with antibiotics and then incubated for 1 hour at 37°C and 5% carbon dioxide in serum-free DMEM/F12 Nutmix (DMEM/F12; Invitrogen, Karlsruhe, Germany) containing 0.1% pronase E (Sigma-Aldrich, Taufkirchen, Germany) in a spinner flask for fine mincing and digestion. After two further washes, overnight enzymatic digestion was performed at 37°C in 0.05% collagenase P (Roche Diagnostics, Mannheim, Germany) in DMEM/F12 media supplemented with 5% fetal calf serum (FCS). Cells were separated by filtration through a 50 μm mesh sieve and washed twice in DMEM/F12 containing 5% FCS and antibiotics. After counting in a Neubauer chamber, cells were seeded in culture flasks at an average density of 2 × 10^4 ^cells/cm^2^, passaged once after 1 week at 90% confluence by trypsin treatment (0.25% trypsin diluted in Versene; Gibco, now part of Invitrogen Corporation, Carlsbad, CA, USA) to eliminate matrix debris, and cultured for maximally one more week in order to obtain sufficient numbers of cells. Media were changed three times a week.

### Flow cytometry

After the adherent cells were detached by trypsin treatment, the cell suspension was washed twice in PBS supplemented with 1% FCS and 0.02% sodium azide (Sigma-Aldrich). A total of 1 × 10^5 ^cells were double-stained for 30 minutes at 4°C with R-phycoerythrin-conjugated anti-CD105 (clone SN6; IgG_1_; Ancell Corporation, Bayport, MN, USA) and fluorescein isothiocyanate-conjugated anti-CD166 monoclonal antibodies (clone 3A6; IgG_1_; Ancell Corporation) or the respective IgG_1 _isotype controls (clone DAK-GO1; Dako, Glostrup, Denmark). The cells were then washed three times with 1% FCS and 0.02% sodium azide, resuspended, and subjected to flow cytometry by using a FACScan and CellQuest software (Becton Dickinson, Heidelberg, Germany). Cells were gated using forward and side scatters to exclude debris and cell aggregates.

### Immunohistochemistry of isolated osteoarthritis and normal chondrocytes

Cells were grown for 2 to 3 days to subconfluence on chamber slides covered with DMEM/F12 supplemented with 5% FCS and antibiotics and then fixed with 4% paraformaldehyde for 10 minutes at room temperature. Non-specific binding sites were blocked with 10% bovine serum albumin for 30 minutes and then the slides were stained for 1 hour at room temperature with 10 μg/mL of the primary antibodies directed against either human CD105 (clone SN6; IgG1; Acris, Hiddenhausen, Germany) or human CD166 (clone 3A6; IgG1; Acris). Mouse IgG_1 _(clone DAK-GO1; Dako) served as an isotype control. Subsequently, CD105-stained cells or isotype control slides were incubated with Alexa Fluor 488-conjugated goat anti-mouse antibody (10 μg/mL). CD166-stained cells and the respective isotype controls were incubated with Alexa Fluor 594-conjugated goat anti-mouse antibody (10 μg/mL). After incubation for 1 hour at room temperature, remaining free binding sites on the first primary antibody were saturated with unlabeled goat anti-mouse IgG (Sigma-Aldrich) (10 μg/mL) for 30 minutes at room temperature. After incubation with the second primary antibody to human CD105 or human CD166 for 1 hour at room temperature, secondary antibody was applied as described above. All respective isotype controls were negative. Cell nuclei were stained with DAPI (4',6-diamidino-2-phenylindole) and washed twice in PBS; subsequently, the chambers were removed, and Prolong antifade reagent (Molecular Probes, now part of Invitrogen Corporation) was applied to the coverslips to prevent photobleaching. Fluorescence-labeled cells were visualized and photographed by using a fluorescence microscope (Axiocam MRm; Carl Zeiss, Oberkochen, Germany) and Axiovision software to create overlay micrographs of the individual channels. Semiquantitative analysis was performed by counting three chamber sectors per patient (magnification 100×; total of approximately 300 cells) for the identification of CD105^+^, CD166^+^, and CD105^+^/CD166^+ ^cells.

### Immunohistochemistry of osteoarthritis and normal cartilage

For immunohistochemical labeling, 4-μm-thick tissue sections were mounted on superfrost plus slides (Menzel, Braunschweig, Germany). After deparaffinization in xylene for 30 minutes, sections were rehydrated through a gradient with decreasing proportions of ethanol. To expose the CD166 molecules, different protocols for antigen retrieval were tested (for example, enzymatical digestion of matrix components by chondroitinase ABC, pronase E, trypsin, collagenase P, or proteinase K). Finally, a digest with proteinase K (code S3004; Dako) for 15 minutes turned out to be most suitable since it led to a sufficient degradation of the interfering cartilage matrix while keeping the sensitive protein structure of the CD166 molecule intact. Endogenous peroxidase activity was blocked by 3% hydrogen peroxide in methanol for 15 minutes. The sections were then blocked for 5 minutes with a universal blocking reagent (Ultra UV Block, TA-125UB; Lab Vision Corporation, now part of Thermo Fisher Scientific Inc., Fremont, CA, USA) and incubated overnight at 4°C with unlabeled primary antibody to human CD166 (10 μg/mL; rabbit polyclonal; Santa Cruz Biotechnology, Inc., Santa Cruz, CA, USA). Normal rabbit IgG instead of the primary antibody was used in negative controls. To reduce background by non-specifically bound primary antibodies, sections were treated for 10 minutes with 1 M NaCl in 0.05 M Tris-buffer pH 7.0. In the next step, binding was detected by incubating the sections for 1 hour with a complex of goat anti-rabbit antibody coupled via polymers to horseradish peroxidase (UltraVision Detection System; Thermo Fisher Scientific, Inc.) in accordance with the instructions of the manufacturer. Using a secondary antibody with a polymer-coupled detection system turned out to be superior to a signal-enhancing system based on biotin in terms of a clearly lower background.

All antibodies were diluted in PBS containing 5% bovine serum albumin. The signal was developed by incubation with hydrogen peroxide and DAB (diaminobenzidine tetrahydrochloride) chromogen. The sections were washed with PBS between the different incubation stages, and all steps were performed at room temperature unless otherwise stated. Sections were counterstained with hematoxylin, mounted with aquatex (Merck, Darmstadt, Germany), and examined by light microscopy.

### Immunomagnetic separation of CD166^+/- ^cells

Expanded OA chondrocytes (passage 1) were detached from culture flasks by trypsin treatment and characterized by fluorescence-activated cell sorting (FACS) as above; 1 × 10^7 ^cells was then placed in a tube containing 10 mL of DMEM/F12 supplemented with 5% FCS and antibiotics. Dynal Magnetic goat anti-mouse IgG beads (Invitrogen, Karlruhe, Germany) (5 particles per cell) were washed with PBS and resuspended in sterile DMEM/F12 supplemented with 5% FCS and antibiotics. The specific monoclonal anti-CD166 antibodies (1 μg/1 × 10^6 ^cells; clone 3A6; Acris) were added to the resuspended goat anti-mouse IgG beads and incubated for 20 minutes at 4°C with slight agitation. The goat anti-mouse IgG beads/anti-CD166 complexes were washed three times with PBS, resuspended in 1 mL of sterile medium, and added to the prepared cell suspension. After incubation for 20 minutes at 4°C with agitation, the mixture was subjected to magnetic separation for 10 minutes. The supernatant containing the CD166^- ^cells was carefully collected, and the pellet of CD166^+ ^cells was resuspended in DMEM/F12 with 5% FCS/antibiotics. In the case of subsequent expansion, antibody-microbead complexes were enzymatically detached from CD166^+ ^cells by chymopapain treatment for 20 minutes at 4°C (10 U/10^6 ^cells; Sigma-Aldrich).

The CD166^+ ^and CD166^- ^cells were either immediately analyzed by FACS or first cultured for up to three passages to reach the appropriate cell numbers for analysis of their multi-lineage potential in differentiation assays and then analyzed by FACS as above.

### Cell differentiation assays

For adipogenic differentiation, CD166^+ ^and CD166^- ^cells from patients with OA were plated at a density of 10,000 cells/cm^2 ^and, 3 days after reaching confluence, stimulated for 15 days with high-glucose DMEM (Invitrogen Corporation) (4,500 mg/L D-glucose) containing 5% human serum and adipogenic supplements: 1 mM dexamethasone, 0.2 mM indomethacin, and 0.5 mM 3-isobutyl-1-methylxanthine (all Sigma-Aldrich) and 10 mg/mL insulin (NovoNordisk, Mainz, Germany). Intracellular lipid droplets in adipogenic cultures were visualized by using oil red O (Sigma-Aldrich).

For osteogenic differentiation, CD166^+ ^and CD166^- ^cells were also plated at a density of 10,000 cells/cm^2 ^in culture dishes and confluent monolayers were cultured for 28 days with low-glucose DMEM (1,000 mg/L D-glucose) containing 5% human serum and osteogenic supplements (0.1 mM dexamethasone, 50 mM L-ascorbic acid-2-phosphate, and 10 mM β-glycerophosphate; all Sigma-Aldrich). Medium was changed every other day. Osteogenic differentiation was assessed histochemically by analyzing alkaline phosphatase activity with sigma fast BCIP/NBT (5-bromo-4-chloro-3-indolyl phosphate/nitro blue tetrazolium) (Sigma-Aldrich).

Chondrogenic differentiation of CD166^+ ^and CD166^- ^cells (passage 3 or 4) was performed under serum-free conditions in high-density pellet cultures (250,000 cells per pellet) as described previously [19]. Chondrogenesis was induced by adding 10 ng/mL TGF-β3 (Peprotech, Hamburg, Germany). The medium was changed every 2 to 3 days, and cells were maintained for up to 28 days. Chondrogenic differentiation was assessed by embedding micro-masses in OCT compound, freezing, and cryosectioning (6 mm). Sections were stained with Alcian blue (Roth, Karlsruhe, Germany) to detect charged molecules such as proteoglycans. Controls for the three differentiation experiments were performed by omitting the respective adipogenic, osteogenic, or chondrogenic supplements.

### Statistics

Results were expressed as mean ± standard error of the mean. Correlations between the respective percentages of CD166^+ ^cells in FACS and immunohistochemical staining in individual samples were analyzed by using the one-tailed Spearman rank test (since a negative correlation between the parameters was not expected) and SPSS 12.0 software (SPSS, Inc., Chicago, IL, USA).

## Results

### Expressions of CD105 and CD166 on isolated chondrocytes from osteoarthritis and normal cartilage

**Figure 2:**
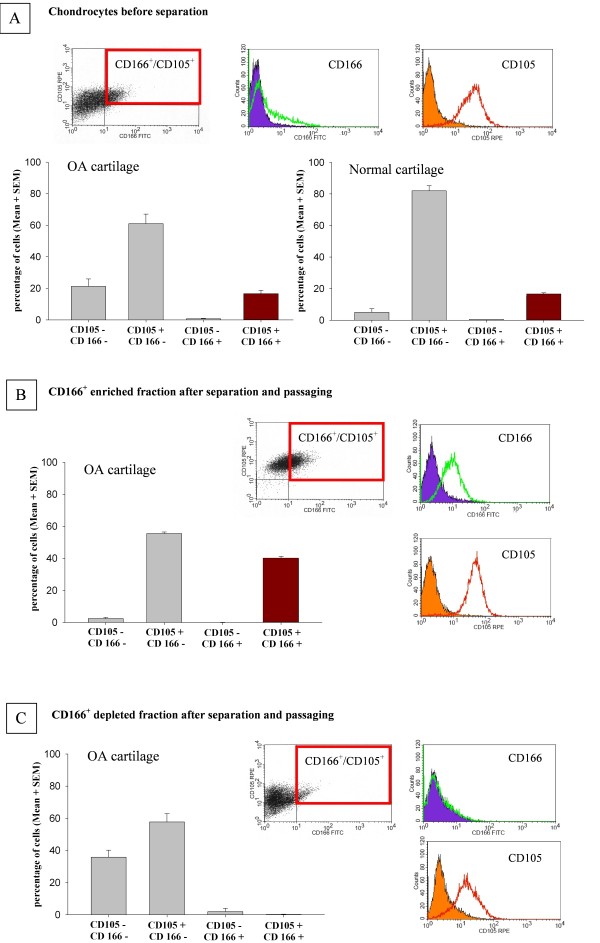
**FACS analysis of CD105 and CD166 expressions on isolated normal and osteoarthritis (OA) chondrocytes and enrichment of CD166**^**+ **^**OA cells after immunomagnetic separation**. Enzymatically isolated, non-separated OA (*n *= 11 patients) and normal (*n *= 3) chondrocytes were subjected to FACS analysis after initial *in vitro *culture **(a)**. After magnetic separation of CD166^+ ^cells, both the positive **(b) **and the negative **(c) **fractions were studied for the expressions of CD105 and CD166. The bar charts show the mean ± standard error of the mean (SEM) for all analyzed patients; for each section, a representative example of the FACS measurement is included as a dot plot diagram, in which CD105^+^/CD166^+ ^cells are located in the upper right quadrant. In addition, the relative expressions of CD105 and CD166 (colored lines) and the corresponding isotype controls (filled graphs) are shown in histograms. FACS, fluorescence-activated cell sorting; FITC, fluorescein isothiocyanate; RPE, R-phycoerythrin.

FACS analysis of CD105 and CD166 expression on the surface of chondrocytes from OA patients (*n *= 11)/normal donors (*n *= 3) showed that 21.3% ± 4.7%/5.1% ± 3.9%, respectively, of the cells were double-negative for CD105 and CD166, 61.0% ± 6.1%/82.4% ± 3.0% were single-positive for CD105, and only 0.7% ± 0.3%/0.5% ± 0.2% were single-positive for CD166 (Figure 2a). Interestingly, 16.7 ± 2.1%/15.3 ± 2.3% of the analyzed cells were double-positive for CD105 and CD166 (Figure 2a), indicating a high percentage of MPCs in adult OA and normal cartilage.

**Figure 3:**
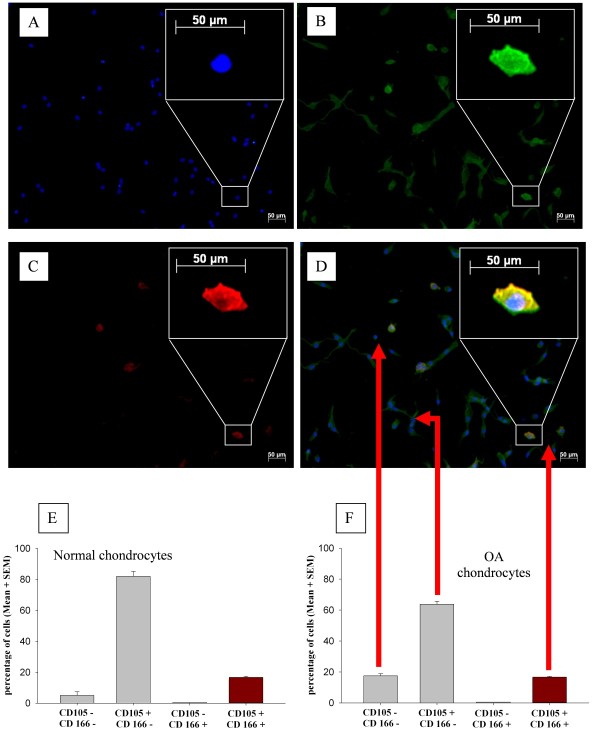
**Immunohistochemical staining of normal and osteoarthritis (OA) chondrocytes after isolation and initial adherence**. Representative images of an OA chondrocyte sample are shown in **(a-d)**; staining results for normal chondrocytes were identical (not shown). Nuclei were visualized with DAPI (4',6-diamidino-2-phenylindole) (a). Cell membranes were double-stained for CD105 by using an Alexa Fluor 488-coupled secondary antibody and for CD166 by using an Alexa Fluor 594-coupled secondary antibody and analyzed by fluorescence microscopy. Co-expression of CD105 and CD166 was identified after computed superimposition of fluorescence signals from both channels. Almost all cells are CD105^+ ^(b), whereas only a few cells express CD166 (c); but in all cases, this is accompanied by the co-expression of CD105 (d). In addition, the results of the quantitative analysis of normal **(e) **and OA **(f) **chondrocytes are shown. Magnifications: 100 ×, 400 × (insets). SEM, standard error of the mean.

Immunohistochemical staining of adherent OA (Figure 3a-d,f) and normal (Figure 3e) chondrocytes basically confirmed these results; that is, 9.3 ± 1.4%/6.2 ± 2.3%, respectively, of the cells were double-negative for CD105 and CD166, 77.5% ± 1.9%/88.4% ± 3.2% were single-positive for CD105, and none was single-positive for CD166. Also, in this analysis, a large percentage of cells (that is, 13.2% ± 0.9%/11.7% ± 2.1%) were double-positive for CD105 and CD166 and thus presumably MPCs (Figure 3). The Spearman rank test indicated that there was a significant positive correlation between the results for FACS and immunohistochemical staining in individual samples from OA patients (*n *= 6, rho = 0.771, *P *= 0.036), further underlining the consistency of the data.

### Enrichment of CD166^+ ^cells from osteoarthritis patients for multi-lineage assays

**Figure 4:**
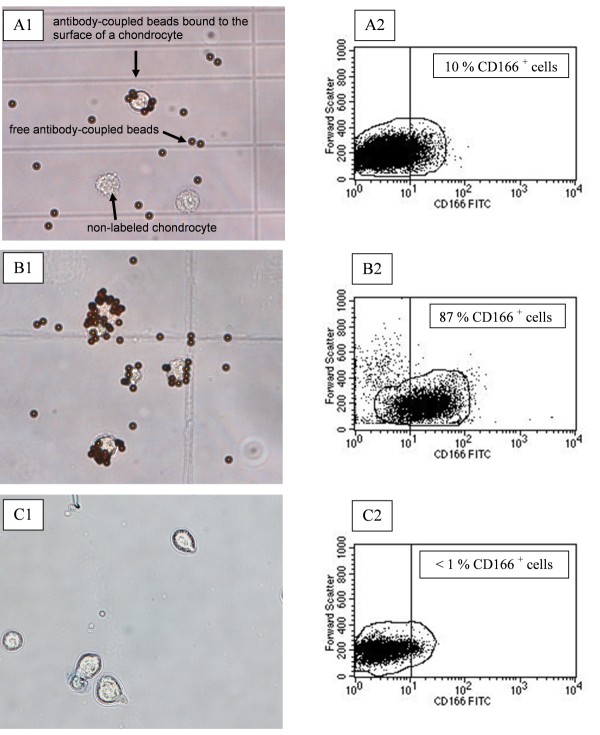
**Microscopic images and corresponding FACS analysis of chondrocytes before and after immunomagnetic separation**. After incubation with bead-coupled antibodies to the surface antigen CD166, both membrane-bound and free magnetic anti-CD166 particles are visible, as are non-labeled chondrocytes **(a1)**. In the positive fraction after separation, only magnetobead-covered CD166^+ ^cells and free magnetobeads are present **(b1)**. In the negative fraction, only CD166^- ^cells without magnetobeads are present; also, no free magnetobeads are present **(c1)**. These results were confirmed by FACS analysis, demonstrating an enrichment of CD166^+ ^cells from 10% before **(a2) **to approximately 87% after **(b2) **separation, whereas the negative fraction contained only a very low percentage of CD166^+ ^cells **(c2)**. FACS, fluorescence-activated cell sorting; FITC, fluorescein isothiocyanate.

After incubation with bead-coupled antibodies to CD166, both magnetobead-coupled cells and magnetobead-free cells were observed; in addition, excess magnetobeads were present (Figure 4a1). In contrast, microscopic analysis of the CD166^+ ^fraction after magnetic separation showed that close to 100% of the cells carried magnetobeads on their surface (Figure 4b1), whereas there were no bead-coupled cells in the CD166^- ^fraction, pointing to a high efficacy of the separation (Figure 4c1). To confirm the microscopic evaluation, re-analysis experiments of the two different fractions by FACS immediately after separation were performed. For this purpose, the routinely used enzymatic detachment of the antibody-microbead complexes from CD166^+ ^cells was omitted since it causes removal of all cell surface molecules for a period of several days. FACS analysis demonstrated an enrichment of CD166^+ ^cells from 10% before up to approximately 87% after separation (Figure 4a2,b2). The negative fraction contained less than 1% CD166^+ ^cells (Figure 4c2).

To obtain sufficient numbers of cells for the multi-lineage assays, CD166^+ ^and CD166^- ^cells were expanded for an additional two or three passages. FACS analysis immediately before the multi-lineage assay showed that approximately 40% of the cells in the positive fraction were still CD166^+ ^and only approximately 2% of the cells in the negative fraction were CD166^+^. This indicates that, despite the expansion, considerable enrichment of CD166^+ ^cells was achieved (approximately 20-fold) (Figure 2b,c).

### Multi-lineage differentiation assay

**Figure 5:**
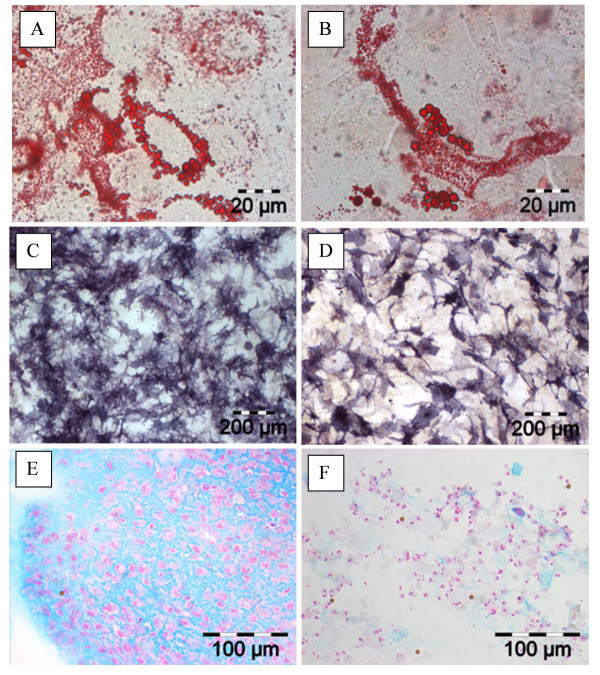
**Cell differentiation studies with CD166**^**+**^**-enriched and -depleted chondrocytes**. Isolated osteoarthritis chondrocytes were immunomagnetically enriched **(a,c,e) **or depleted **(b,d,f) **for CD166^+ ^cells and subjected to adipogenic, osteogenic, and chondrogenic differentiation. CD166^+^-enriched as well as CD166^+^-depleted cell populations differentiate to adipocytes after culture in adipogenic medium, as detected by oil red O staining (a,b). After cultivation with osteogenic medium, both cell populations demonstrate differentiation toward osteoblasts observable as positive alkaline phosphatase activity within the cells (c,d). However, this effect is more pronounced in cells previously enriched for CD166^+ ^chondrocytes (c). Pellet cultures of cells that underwent chondrogenic differentiation are visualized by Alcian blue staining of newly deposited proteoglycans (e,f). Exclusively, the population with CD166^+^-enriched cells shows a clear chondrogenic phenotype (e).

In the case of adipogenesis, oil red staining revealed that CD166^+ ^as well as CD166^- ^cells from patients with OA differentiated toward adipocytes without clear differences between the two groups (Figure 5a,b). Induction of the osteogenic lineage, as assessed by the detection of alkaline phosphatase, was more pronounced in CD166^+ ^cells, indicating more progenitor cells in CD166^+ ^than CD166^- ^cells (Figure 5c,d). As for chondrogenesis, only the pellet cultures of CD166^+ ^cells showed a clear deposition of extracellular matrix as detected by Alcian blue staining for sulphated proteoglycans. In contrast, the CD166^- ^cell pellets were not characterized by the formation of cartilage-specific matrix components (Figure 5e,f).

No signs of osteogenesis were observed in the chondrogenic micromass cultures of CD166^+ ^cells up to 3 weeks, as shown by strong expression of collagen type II and weak or absent expression of collagen type X and alkaline phosphatase (Supplemental Table S1 in Additional file 1). The same was true in chondrocyte micromass culture without chondrogenic supplements, in which there was no RNA upregulation of alkaline phosphatase, collagen type X, or Runx-2 [20] (Supplemental Figure S1 in Additional file 1).

### Immunohistological localization of CD166^+ ^chondrocytes within the cartilage matrix

**Figure 6:**
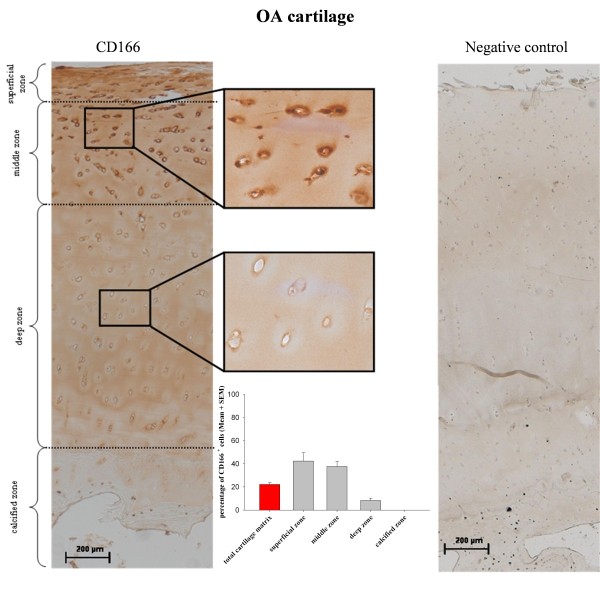
**Distribution of CD166**^**+ **^**chondrocytes within the osteoarthritis (OA) joint cartilage matrix**. CD166^+ ^cells, characterized by an intense brown staining of the cell membrane and pericellular matrix, are localized primarily in the superficial and middle cartilage zones of OA cartilage. In contrast, almost no CD166^+ ^cells are in the deep and calcified cartilage zones. The corresponding isotype control of the immunohistochemical mesenchymal progenitor cell staining shows an absence of a positive signal, supporting the specificity of the above-named CD166 staining. Magnifications: 100 × (large micrograph), 400 × (insets). Results of semiquantitative analysis of cartilage samples from six patients with OA are expressed as mean ± standard error of the mean (SEM).

**Figure 7:**
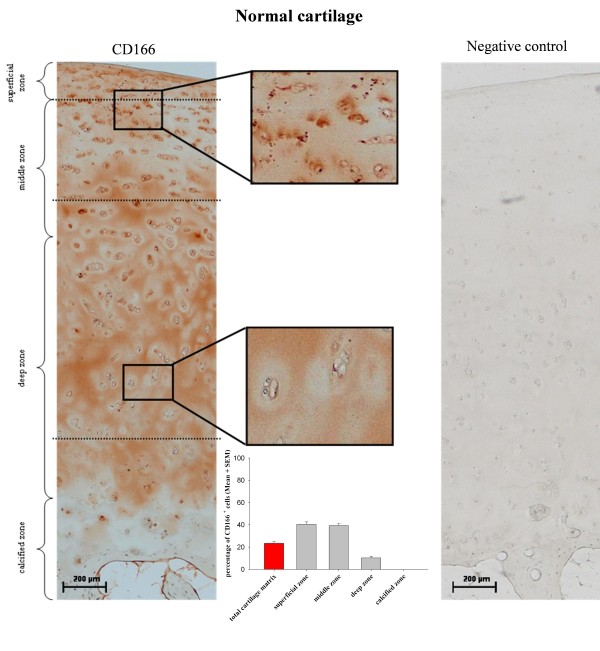
**Distribution of CD166**^**+ **^**chondrocytes within the normal joint cartilage matrix**. CD166^+ ^cells within normal cartilage matrix show a distribution pattern similar to that of osteoarthritic cartilage (shown in Figure 6), and the absence of a positive staining signal in the corresponding isotype control proves the specificity of the CD166 staining. Magnifications: 100 × (large micrograph), 400 × (insets). Results of semiquantitative analysis of cartilage samples from three normal donors are expressed as mean ± standard error of the mean (SEM).

Besides quantifying the relative percentage of CD166^+ ^chondrocytes by immunofluorescence, analyzing the zonal distribution of these cells within the matrix of normal and OA cartilage was of particular interest. Owing to the inherent autofluorescence properties of cartilage matrix components and chondrocytes (data not shown), preliminary tests with fluorescence-based detection systems did not lead to the desired result. In contrast, the detection of cell membrane proteins embedded in the dense extracellular matrix was successfully accomplished by using a modified protocol by Ozbey and colleagues [21] (see Materials and methods). Interestingly, the distribution of the CD166^+ ^cells in OA and normal samples was not uniform but was clearly zonal (Figures 6 and 7). In both OA and normal cartilage, CD166^+ ^cells were almost exclusively located in the superficial and middle cartilage zones.

As expected for a cell surface molecule, the staining signal was associated mainly with chondrocyte membranes and pericellular areas. Specifity of the staining was supported by the absence of a positive signal in the isotype control (Figures 6 and 7, right panels).

Semiquantitative analysis of stained sections revealed percentages of 22.1% ± 1.7% CD166^+ ^chondrocytes within the total cartilage matrix of OA patient samples (*n *= 6) (Figure 6) and 23.6% ± 1.4% in the case of normal cartilage (*n *= 3) (Figure 7). Thus, the FACS and immunhistochemical results obtained for isolated chondrocytes were confirmed. The percentages of CD166^+ ^chondrocytes in the superficial cartilage zone of OA and normal cartilage were 42.4% ± 7.2%/40.4% ± 2.3%, respectively; 37.8% ± 4.4%/39.4% ± 1.6% in the middle zone; and 8.4% ± 2.0%/10.2% ± 1.6% in the deep zone, whereas no CD166^+ ^cells were detected in the calcified zone of either OA or normal cartilage (Figures 6 and 7).

## Discussion

### Abundance of mesenchymal progenitor cells in osteoarthritis and normal cartilage

High percentages of CD105^+^/CD166^+ ^cells were observed by FACS in OA/normal cartilage (16.7 ± 2.1%/15.3 ± 2.3%, respectively), by immunohistochemical staining of isolated cells (13.2% ± 0.88%/11.7% ± 2.1%), and, most importantly, by *in situ *immunodetection (22.1% ± 1.7/23.6% ± 1.4%). Since this is an established marker combination for MPCs [5], considerable numbers of these multi-potent cells appear to be present in OA and normal cartilage.

The present percentages are higher than those in previous reports (2% to 12% [4] or approximately 7.5% [5], respectively), thus suggesting a yet unexplored reserve capacity for regeneration, especially in OA cartilage. However, the chondrocytes in previous studies were analyzed either immediately following enzymatic harvest [4] or after short-term primary culture (P0) [5,22]. It is therefore possible that the initial expansion of MPCs for 2 weeks in the present study led to a relative increase of the cells and an overestimation of the percentage of MPCs in native OA cartilage [4]. This is somewhat unlikely since the percentage of CD166^+ ^cells decreased from approximately 87% immediately following separation to approximately 40% after culture for approximately 3 weeks. Also, the percentage of CD166^+ ^cells in OA and normal cartilage sections was comparable to or even higher than that in FACS or staining of isolated cells in culture. On the other hand, heterogeneity among patient cohorts in different studies or differences in the preparation techniques (such as the inclusion/exclusion of the calcified cartilage, which represents up to 30% of the total cartilage [23]) may have influenced the results.

In the present study, in contrast to previous reports [5,14], the percentage of MPCs did not differ in OA and normal cartilage. One potential explanation could be that, in the present study, cartilage samples were exclusively obtained from areas with mild to moderate macroscopic alterations - which may contain higher numbers of vital MPCs. However, no differences concerning the percentages of CD105^+^/CD166^+ ^MPCs in normal-appearing and degraded OA cartilage have been previously observed [5]. In addition, severe alterations of the metabolic functions in OA chondrocytes (and presumably of MPCs) do not seem to be restricted to the lesioned areas but appear to affect the complete cartilage in OA joints [24]. In view of the high percentages of MPCs in OA cartilage and the fact that at least bone marrow-derived MSCs from OA patients do not show a lower chondrogenic potential than MSCs from normal donors [25,26], future studies may have to explicitly focus on exploiting the regenerative potential of OA MPCs.

### Suitability of CD166 as a sole biomarker for mesenchymal progenitor cells in cartilage

It is believed that MPCs in cartilage co-express the markers CD105 and CD166 and that there are no CD166^- ^MPCs. Since approximately 99% of the MPCs in the present study co-expressed CD105 and CD166, CD105 may be dispensable and CD166 may suffice to unequivocally identify and enrich MPCs from cartilage. This is in line with the fact that CD166 acts as a central mediator in cell adhesion, growth, and migration [27]. Indeed, CD166^+ ^cells from OA patients were somewhat more potent in osteogenesis and were the only cells capable of undergoing chondrogenesis under the respective culture conditions. The lack of increased capacity of CD166^+ ^cells for adipogenesis may be due to the advanced stage of OA in the cartilage donors [28]. On the other hand, the CD166^- ^chondrocytes showed some limited multipotency in the present assays, indicating that there may also be CD166^- ^MPCs in cartilage [5]. Whether the minute fraction of CD166^+^/CD105^- ^cells in cartilage also carries MPC features needs to be further analyzed.

### Zonal distribution of CD166^+ ^mesenchymal progenitor cells in the cartilage matrix

CD166^+ ^chondrocytes were almost exclusively located in the superficial and middle zones of OA and normal cartilage. This zonal preference confirms a previous report in normal rat cartilage [10] and extends the findings to human OA and normal cartilage. Indeed, the surface zone seems to be of central importance for the growth of articular cartilage in young animals [29] and presumably relies on a progenitor cell population located in this zone [30]. Also, in rat and cow, these progenitor cells carry specific stem cell markers such as Notch-1 and CD166 [10,21], showing the high consistency of this zonal enrichment among species. The MPC marker CD166 could thus serve as a valuable tool to improve matrix-based cartilage regeneration strategies concerning their specific three-dimensional design, the addition of cell-recruiting bioactive factors, and/or optimized seeding with cartilage-derived MPCs [28,31-33]. This is of special interest since newer repair strategies using cell-free matrices in combination with microfracturing aimed at mobilizing resident multi-potent MPCs from the bone marrow have shown the feasibility of such concepts and have entered clinical practice [34-37].

## Conclusions

The present study suggests that CD166 is a suitable biomarker for the identification and localization of resident MPCs with high chondrogenic potential in human articular cartilage. In addition, the percentage of MPCs in OA and normal cartilage is substantially higher than previously reported, indicating a yet unexplored reserve capacity for regeneration in the event of chondral degradation. The knowledge about their preferred localization in the superficial and middle zones of OA and normal cartilage may open new therapeutic opportunities concerning both matrix-based cell-containing implants and cell-free *in situ *regeneration.

## Abbreviations

DMEM/F12 Nutmix: Dulbecco's modified Eagle's medium/F12 Nutmix; FACS: fluorescence-activated cell sorting; FCS: fetal calf serum; IgG: immunoglobulin G; MPC: mesenchymal progenitor cell; MSC: mesenchymal stem cell; OA: osteoarthritis; PBS: phosphate-buffered saline; TGF: transforming growth factor.

## Competing interests

The authors declare that they have no competing interests.

## Authors' contributions

DP and SA contributed to study conception and design, acquisition of data, analysis and interpretation of data, and drafting or critical revision of the manuscript or both. CK and RWK contributed to study conception and design, analysis and interpretation of data, and drafting or critical revision of the manuscript or both. SL contributed to acquisition of data, analysis and interpretation of data, and drafting and critical revision of the manuscript. SR contributed to acquisition of data and critical revision of the manuscript. ME contributed to acquisition of data and to critical revision of the manuscript. All authors have read and approved the final manuscript.

## Supplementary Material

Additional file 1 **Additional data concerning the differentiation status of chondrocyte micromasses**. The file contains data from the immunohistological characterization of CD166^+^-enriched micromasses after culture in chondrogenic medium as well as gene expression profiles of selected marker genes for hypertrophy and/or osteogenic lineage development in human chondrocyte high-density micromasses. Click here for file
