# Supplementary material for: Relative percentage and zonal distribution of mesenchymal progenitor cells in human osteoarthritic and normal cartilage
Source: Arthritis Res Ther. 2011 Apr 15;13(2):R64. doi: 10.1186/ar3320 (PMC3132059; doi:10.1186/ar3320)
Supplement: Additional file 1 — Additional data concerning the differentiation status of chondrocyte micromasses. The file contains data from the immunohistological characterization of CD166+-enriched micromasses after culture in chondrogenic medium as well as gene expression profiles of selected marker genes for hypertrophy and/or osteogenic lineage development in human chondrocyte high-density micromasses. [file ar3320-S1.DOC]

**Supplementary material**

**Supplementary Materials & Methods**

**Immunohistological characterization of CD166^+^-enriched micromasses after culture in chondrogenic medium**

The micromass pellets were harvested after 1–3 weeks of culture for immunohistochemical detection of collagens type II and X (incl. the isotype controls). Alkaline phosphatase activity was histochemically assessed using a BCIP/NBT substrate (Sigma-Aldrich).

**Hypertrophy/osteogenic marker gene profiles in chondrocyte micromasses**

Gene expression profiles of selected marker genes for hypertrophy and/or osteogenic lineage development in human chondrocyte high-density micromasses were obtained from a previously published microarray data set [20]. In brief, native human chondrocytes from 3 normal donors were pooled for each of the 2 experimental samples and analyzed directly after enzymatic digestion of the cartilage matrix or following culture in micromasses for 3 or 6 weeks. Samples were subjected to RNA isolation and subsequent analysis using oligonucleotide microarrays HG-U133A (Affymetrix). Gene expression raw data were processed and normalized by robust multiarray analysis. Selected marker gene profiles were visualized by hierarchical cluster analysis with normalized gene expression values and the Pearson correlation distance, performed with the Genesis 1.1.3 software. Differential expression was accepted at a fold-change of at least 2 or -2.

**Supplementary results**

**Expression of chondrogenic and osteogenic markers in** **CD166^+^-enriched micromasses**

Immunohistological analysis of the CD166^+^-enriched chondrocytes (cultured as micromass pellets in chondrogenic medium) revealed an induction of the cartilage-specific collagen type II after 1, 2 and, in particular, 3 weeks, demonstrating a stable chondrogenic phenotype of the cells (Supplementary Table 1). Protein expression of collagen type X, a marker for hypertrophy/osteogenic differentiation, clearly decreased from 1 to 3 weeks of culture in chondrogenic medium – thus demonstrating the absence of indications for such processes. This finding was further supported by the lack of alkaline phosphatase activity after 3 weeks.

| **Protein/activity** | **Duration of micromass culture** | | |
| --- | --- | --- | --- |
|  | **1 week** | **2 weeks** | **3 weeks** |
| **IgG isotype control** | neg. | neg. | neg. |
| **collagen type II** | ++ | ++ | +++ |
| **collagen type X** | +++ | + | + |
| **alkaline phosphatase** | ND | ND | neg. |

**Supplementary Table 1: Semiquantitative analysis of immunohistological staining for collagen types II and X, as well as histochemical detection of alkaline phosphatase activity in CD166^+^-enriched micromasses.** neg. = negative; + = weak positive staining; ++ = moderate positive staining; +++ = strong positive staining; ND = not determined.

**Hypertrophy/osteogenic marker gene profiles in chondrocyte micromasses**

Hierarchical clustering of hypertrophy and/or osteogenic differentiation expression profiles showed that the micromasses cultured in vitro for 3 or 6 weeks groups are closely related to each other (Supplementary Fig. 1). There was no evidence for an upregulation of osteogenic gene markers such as alkaline phosphatase, collagen type X α1 or Runx2.


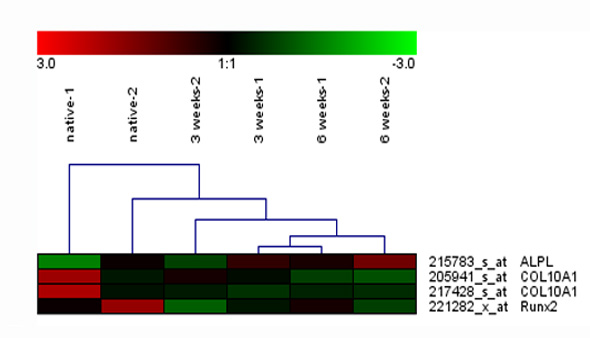


**
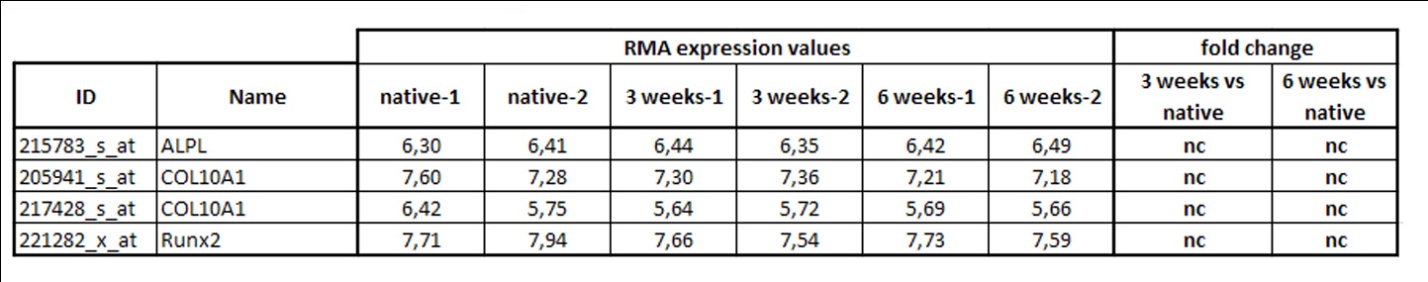
**

**Supplementary Figure 1: Hypertrophy/osteogenic marker gene profiles in chondrocyte micromasses.** Hierarchical clustering and robust multiarray analysis (RMA) expression values of typical hypertrophy/osteogenic marker genes in chondrocyte micromasses showed no evidence for hypertrophy or osteogenic differentiation. Strongly expressed genes are given in red and weakly repressed genes are given in green. nc - no change (< 2 or -2-fold), ALPL – alkaline phosphatase (bone/liver/kidney form), COL10A1 – collagen type X α1 chain, Runx2 – runt-related transcription factor 2.
